# [Retracted] Total flavonoids suppress lung cancer growth via the COX‑2‑mediated Wnt/β‑catenin signaling pathway

**DOI:** 10.3892/ol.2024.14212

**Published:** 2024-01-08

**Authors:** Lei Han, Shu Fang, Guangtao Li, Minghuan Wang, Renzhi Yu

Oncol Lett 19: 1824–1830, 2020; DOI: 10.3892/ol.2020.11271

Following the publication of this paper, it was drawn to the Editor's attention by a concerned reader that the cell apoptotic data shown in Fig. 4B and the histological data shown in Fig. 4C on p. 1828 were strikingly similar to data appearing in different form in other articles written by different authors at different research institutes, which had either already been published or were under consideration for publication at around the same time. Owing to the fact that the contentious data in the above article had already been published prior to its submission to *Oncology Letters*, the Editor has decided that this paper should be retracted from the Journal. The authors were asked for an explanation to account for these concerns, but the Editorial Office did not receive a reply. The Editor apologizes to the readership for any inconvenience caused.

